# The first identification of genomic loci in plants associated with resistance to galling insects: a case study in *Eucalyptus* L'Hér. (Myrtaceae)

**DOI:** 10.1038/s41598-018-20780-9

**Published:** 2018-02-02

**Authors:** Miaomiao Zhang, Changpin Zhou, Zhijiao Song, Qijie Weng, Mei Li, Hongxia Ji, Xiaoyong Mo, Huanhua Huang, Wanhong Lu, Jianzhong Luo, Fagen Li, Siming Gan

**Affiliations:** 1 State Key Laboratory of Tree Genetics and Breeding, Chinese Academy of Forestry, Xiangshan Road, Beijing, 100091 China; 2 Key Laboratory of State Forestry Administration on Tropical Forestry Research, Research Institute of Tropical Forestry, Chinese Academy of Forestry, Longdong, Guangzhou, 510520 China; 3 College of Forestry, South China Agricultural University, 284 Block, Wushan Street, Guangzhou, 510642 China; 4 Baoshan University, Yuanzheng Road, Baoshan, 678000 China; 5 Guangdong Academy of Forestry, Longdong, Guangzhou, 510520 China; 6 China Eucalypt Research Centre, Zhanjiang, 524022 China

## Abstract

Genomic loci related with resistance to gall-inducing insects have not been identified in any plants. Here, association mapping was used to identify molecular markers for resistance to the gall wasp *Leptocybe invasa* in two *Eucalyptus* species. A total of 86 simple sequence repeats (SSR) markers were screened out from 839 SSRs and used for association mapping in *E*. *grandis*. By applying the mixed linear model, seven markers were identified to be associated significantly (*P* ≤ 0.05) with the gall wasp resistance in *E*. *grandis*, including two validated with a correction of permutation test (*P* ≤ 0.008). The proportion of the variance in resistance explained by a significant marker ranged from 3.3% to 37.8%. Four out of the seven significant associations in *E*. *grandis* were verified and also validated (*P* ≤ 0.073 in a permutation test) in *E*. *tereticornis*, with the variation explained ranging from 24.3% to 48.5%. Favourable alleles with positive effect were also mined from the significant markers in both species. These results provide insight into the genetic control of gall wasp resistance in plants and have great potential for marker-assisted selection for resistance to *L*. *invasa* in the important tree genus *Eucalyptus*.

## Introduction

There are approximately 132,930 insect species around the world that can infect plant tissues and induce tumor-like gall formation^1^. Gall-inducing insects (gallers) belong principally to the orders Diptera (mainly family Cecidomyiidae), Hymenoptera (mainly Cynipidae), Hemiptera and Thysanoptera^1,2^. Although gall tissues provide a protected nutrient-rich and favourable microenvironment to the gallers^3^, many of the gallers act as parasites to plants and affect adversely host growth, thereby posing serious agricultural and forestry threats^4,5^. For instance, the leave- or root-galling phylloxera (*Daktulosphaira vitifoliae* Fitch) devastated the grape (*Vitis vinifera* L.) production and wine industry in Europe in the 1860s and again threatened the viticulture in California in the 1980s^6^. Thus, resistance to galler pests has been a pivotal breeding objective in those crops subject to galling risk.

**Figure 1:**
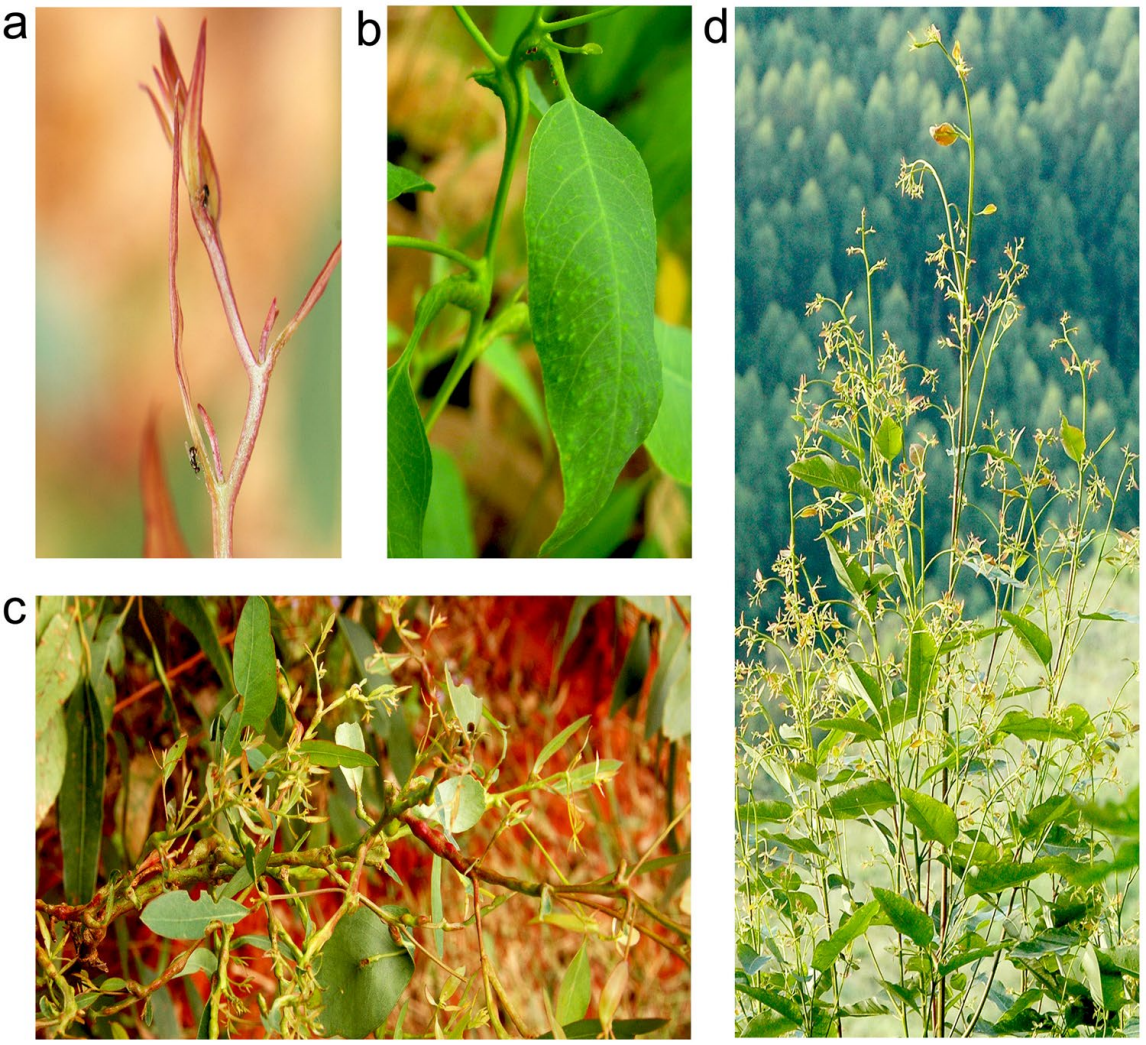
The galling insect *Leptocybe invasa* and its infection sympotoms on *Eucalyptus*. (**a**) *L*. *invasa* adults (~1.2 mm in length) attacking eucalypt shoots. (**b**) Galls on leaf and petiole (*E*. *tereticornis*), (**c**) galls on tender stem and branch (*E*. *grandis*) and (**d**) multiple sprouts (*E*. *grandis*) after *L*. *invasa* infection. Photos were taken by Huanhua Huang.

The woody plant genus *Eucalyptus* L′Hér. (family Myrtaceae) is almost entirely native to the Australian continent and adjacent islands^7^. *Eucalyptus* trees (eucalypts) have been cultivated worldwide for timber, fuel, pulp and paper purposes, with global plantations totaling at more than 21 million ha^8^. In their native range, eucalypts sustain a rich fauna of gall-inducing insects^9^ and are also specific hosts to several gall wasps in the family Eulophidae (Hymenoptera: Chalcidoidea), including *Leptocybe invasa* Fisher & La Salle (Fig. 1) which typically induces bump-shaped galls on the leaf midribs, petioles and stems of young susceptible trees^10^. *L*. *invasa* can cause retardation of host growth and devastating damage to eucalypt nurseries and plantations^11^. Outside the native range of eucalypts, *L*. *invasa* was first observed in Israel in 2000 and spread subsequently over many other regions in Africa, Europe, Asia and South America^10–12^. Biological controls have been attempted using parasitoid wasps like *Quadrastichus mendeli* Kim & La Salle and *Selitrichodes kryceri* Kim & La Salle^13^, however, selection of resistant or less susceptible genotypes has potential in mitigating the damage from the galler insect given the fact that variation in resistance exists among species, provenances, genotypes and/or clonal varieties in *Eucalyptus*^10–12,14–16^.

Exploration of genetic variation related with phenotypic differences will shed insights into the genetic mechanism underlying trait formation and identify valuable markers for selection in breeding schemes. In recent decades, the wealth of molecular markers developed in many plant species has enabled the genetic dissection of phenotypic traits using DNA based approaches, including quantitative trait locus (QTL) mapping, association (or linkage disequilibrium, LD) mapping (AM) and genomic selection^17^. QTL mapping uses bi-parental segregating populations to determine genomic regions influencing complex traits and has proved to be useful in a wide spectrum of plants^18,19^. Genomic selection applies breeding populations to scan genome-scale molecular data for optimal phenotypes and holds great promise for plant breeding efforts^20^. However, both QTL mapping and genomic selection have relatively low resolution with the causative genetic variant, and marker-gene linkage can be lost through recombination in other populations or advanced generations, thus limiting their applications in breeding and gene function studies^17^. In this regard, AM has been proposed as a powerful method for fine mapping because it can take full advantage of LD and historical recombinations in natural populations to identify molecular markers located within the extent of LD decay of a causal or functional genetic variant^19,21,22^. To date, though QTL mapping has resulted in discovery of several genomic loci in plants related with resistance to insects, such as sorghum [*Sorghum bicolor* (L.) Moench] to *Spodoptera frugiperda* J. E. Smith (Lepidoptera: Noctuidae)^23^ and soybean [*Glycine max* (L.) Merr.] to *Aphis glycines* Matsumura (Hemiptera: Aphididae)^24^, few AM studies have involved insect resistance^25^. Specifically, genomic loci related with galler insect resistance have not yet been reported in any plants, and the genetic mechanism underlying such a resistance remains to be clarified.

AM can be performed via genome-wide marker or candidate gene (CG) approaches. For outcrossing species that exhibit great genetic diversity and rapidly decaying LD, e.g. within about 1,500 bp on average in maize (*Zea mays* L.)^26^, a typical genome-wide AM may need tens of millions of markers to be accurately genotyped with numerous individuals, which is still challenging and costly^22^. Consequently, the CG approach has been widely adopted for AM work with outcrossing plants, such as maize^27,28^ and several forest trees^17,29^. However, this approach is inherently limited by the *a priori* choice of CGs which precludes the causal mutations located in nonidentified CGs^21^. Also, the trait variation explained by individual markers (usually single nucleotide polymorphism, SNP) is very low and rarely exceeds 5%^30^. In particular, it is impractical for those traits that no CGs have been discovered. On the other hand, genome-wide LD decay has been revealed to be substantially slower in outcrossing plants (e.g. approximately 3.7–5.7 kb with the largest LD up to 50 kb in *E*. *grandis*^31^) than previous estimates with CGs. More recently, with the rapid development of genomic technologies and resources, genome-wide AM has been attempted using next generation sequencing based SNPs (e.g. maize^32^), microarray-based SNPs [e.g. *Picea glauca* (Moench) Voss^33^] and microsatellites [or simple sequence repeats, SSR; e.g. rice (*Oryza sativa* L.)^34^, *Punica granatum* L.^35^, *Theobroma cacao* L.^36^ and *Ipomoea batatas* L.^37^].

In this study, we used a select set of SSR markers to perform AM in *E*. *grandis* Hill ex Maiden for resistance to the gall wasp *L*. *invasa* and verified the associated SSRs in *E*. *tereticornis* Smith. SSRs have been the choice of markers for AM studies in many selfing^34,38,39^ and outcrossing plants^35–37^. Both *E*. *grandis* and *E*. *tereticornis* are important species in terms of breeding and genomic efforts^40^, and *E*. *grandis* is the second tree genome (after *Populus trichocarpa* Torr. & Gray) to be sequenced^41^. Variation in *L*. *invasa* resistance has been observed in *E*. *grandis*^15^ and also *E*. *tereticornis*^16^. Low population differentiation has been identified in *E*. *grandis* by isozyme markers (*G*_ST_ = 0.12)^42^ and SSRs (*F*_ST_ = 0.037)^43^ and also in *E*. *tereticornis* by SSRs (*F*_ST_ = 0.012)^44^, suggesting a weak population structure that is ideal for AM analyses. Furthermore, though verification of association in additional population(s) is a valuable tool to demonstrate cross-population utility, only a few studies in plants have to date conducted it^17^. The objectives of this study were to (1) detect and verify the marker loci associated with resistance to *L*. *invasa* and (2) identify the favourable alleles for potential use in marker assisted selection in *Eucalyptus*. So far to our knowledge, this is the first report of mapping genomic loci associated with gall-inducing pest resistance in plants.

## Materials and Methods

### Plant materials

A total of 470 individual trees of *E*. *grandis* were sampled as a ‘discovery’ population from a provenance/progeny trial located at Zhaoqing City (112°27′E, 23°03′N), Guangdong Province, China. The trial was laid out following a randomized complete block design, with 32 replicates of single-tree (per family) plots at 2 × 3 m spacing. One to five trees (the first five replicates) were sampled from each of 158 open-pollinated (half-sib) families representing 16 natural seed sources (provenances) across the range of *E*. *grandis* in Australia^43^. A ‘verification’ population of 303 individual trees of *E*. *tereticornis* was sampled from a provenance/progeny trial located at Zhanjiang City (110°05′E, 21°16′N), Guangdong Province, China, which had been planted with four replicates of four-tree (per family) row plots in a randomized complete block design^16^. One to seven trees (the first two replicates) were sampled from each of 77 open-pollinated families from 11 natural provenances in Australia as described earlier^16^. Leaf samples were collected in July 2011 and March 2015 for *E*. *grandis* and *E*. *tereticornis* at ages of 15 and 31 months after planting, respectively. The leaves were stored at −80 °C prior to DNA extraction.

### Assessment of *L*. *invasa* infestation

Natural infestation of *L*. *invasa* was assessed for the *E*. *grandis* trial at age of 15 months when the gall incidence was evident. As different infestation indices were adopted in the literature^11,14,15^, we employed a five-grade criterion based on the number of galls visible on a whole tree as performed similarly by Goud *et al*.^14^, namely, grade 1 = 50 and more galls, grade 2 = 10–49 galls, grade 3 = 9 and less galls, grade 4 = multiple sprouts without galls and grade 5 = no symptom.

For the *E*. *tereticornis* trial, susceptibility by *L*. *invasa* was scored previously at nine months based on the percentage of galled leaves and twigs^16^. The scores were then approximated to the above five-grade criterion depending on infested leaf and twig numbers assuming a mean of two galls per leaf or twig. As the number of galls is strongly positively correlated with mean severity score (based on percentage infestation/100) and proportion of plants infested^12^, such an approximation would provide valid estimation of the infestation grades.

Phenotypic variation in *L*. *invasa* infestation was assessed using the statistical software SAS/STAT^®^ 8.1 (SAS Institute Inc., Cary, NC, USA). Analysis of variance (ANOVA) was conducted only for *E*. *grandis* (based on the relatively complete replicates 1−4) as that of *E*. *tereticornis* had been reported earlier for the whole trial^16^. Narrow-sense heritability $({h}_{i}^{2})$ was calculated as: ${h}_{i}^{2}=1/r\times {\sigma }_{F}^{2}/({\sigma }_{F}^{2}+{\sigma }_{P}^{2}+{\sigma }_{E}^{2})$, where *r* is the coefficient of relationship between the individuals within families (*r* = 0.40 for most open-pollinated families from natural stands of *Eucalyptus*^45^), ${\sigma }_{F}^{2}$ is the among-family variance within provenances, ${\sigma }_{P}^{2}$ is the among-provenance variance, and ${\sigma }_{E}^{2}$ is the residual error variance. Standard error of *h*_*i*_^2^ was estimated using the delta method^46^.

### DNA extraction and SSR marker assay

Genomic DNA was extracted from leaf samples (~300 mg per sample) using a modified cetyltrimethyl ammonium bromide (CTAB) method^47^. DNA concentration and quality were assessed using 1.2% agarose gel electrophoresis and a NanoDrop 2000 spectrophotometer (Thermo Fisher Scientific Inc., Waltham, MA, USA).

A total of 839 genome-wide SSRs as used in Li *et al*.^48^ were initially tested with one *E*. *grandis* DNA sample using routine polymerase chain reaction (PCR) amplification^49^. Those markers (561 SSRs, 66.9%) each resulting in a single clear band in agrose gel electrophoresis were subsequently screened against two sample pools of *E*. *grandis*, namely, resistant pool (four and four samples at grades 5 and 4, respectively) and susceptible pool (eight samples at grade 1). The markers (86 SSRs distributing across the 11 main scaffolds and a small scaffold of *E*. *grandis* genome; Supplementary Fig. S1) that exhibited at least 0.20 of allelic frequency difference between the resistant and susceptible pools were finally selected out for genotyping the ‘discovery’ population of *E*. *grandis*. The SSR genotyping method followed the fluorescein-12-dUTP based procedure as described earlier^49^.

In addition, 25 and 12 putatively neutral genomic SSRs (Supplementary Table S1) were used for population structure analysis in *E*. *grandis* and *E*. *tereticornis*, respectively. These SSRs were previously reported to neither depart significantly from Hardy-Weinberg equilibrium (HWE; *P* < 0.01) nor show outlying between-population differentiation (*F*_ST_) values in *E*. *grandis*^43^ and/or *E*. *tereticornis*^44^.

### Marker polymorphism, linkage disequilibrium (LD) and population structure

For *E*. *grandis*, number of alleles (*N*_A_), observed heterozygosity (*H*_O_), expected heterozygosity (*H*_E_), allele size range (ASR) and polymorphic information content (*PIC*) per SSR marker were estimated with MSA software^50^. LD between the SSRs was evaluated using TASSEL 3.0 software^51^. The determination coefficient (*r*^2^) was used to test the LD pattern with 100,000 permutations.

STRUCTURE 2.3.4 software^52^ was performed to cluster individuals into a number (*K* = 1−16 and 1−11 for *E*. *grandis* and *E*. *tereticornis*, respectively) of genetically homogeneous sub-populations based on an admixture model with correlated allele frequencies between provenances. For each *K* value, the Markov Chain Monte Carlo (MCMC) sampling was replicated with 10 runs^53^ each following 100,000 burn-ins and 100,000 MCMC iterations. The optimal *K* value was determined with the highest Δ*K* method^54^ in STRUCTURE HARVESTER 0.6^55^. The membership coefficient (Q) of each individual generated under the optimal *K* value was used to form the population structure Q matrix. Also, pair-wise kinship coefficients (K matrix) between individuals were estimated using SPAGeDi1-5a software^56^. The Q and K matrices were incorporated into the subsequent association analysis.

### Association mapping and verification

A mixed linear model (MLM) was performed using TASSEL 3.0 software^51^ [file type option ‘Load polymorphism alignment (custom)’] for marker-trait association mapping in *E*. *grandis* and association verification in *E*. *tereticornis*. In order to avoid possible spurious associations, Q and K matrices generated above were incorporated as co-variates (Q + K method). The significant association probability was set at *P* ≤ 0.05. The *R*^2^ value indicated the percentage of phenotypic variance explained by the marker identified. Only markers with allele frequencies of 5% or higher were included in association analysis. Also, the significant associations were further validated with a correction of permutation test (*P* ≤ 0.008 and 0.073 for *E*. *grandis* and *E*. *tereticornis*, respectively). The significant markers were function annotated by BlastX search of their original sequences against NCBI non-redundant protein database (https://blast.ncbi.nlm.nih.gov/Blast.cgi) with a cutoff *E*-value of 10^−5^.

Phenotypic allele effect was estimated in comparison to the average phenotypic value of ‘null allele’ (including the rare alleles with frequency less than 5%)^57^. An allele of positive effect was identified as favourable allele for *L*. *invasa* resistance. The general mean of positive or negative allelic effects was calculated as the average (positive or negative) allelic effect (AAE) of a marker, and its percentage taking account of the average ‘null allele’ phenotypic effect was also calculated^58^.

## Results and Discussion

### *L*. *invasa* resistance variation

The mean value of *L*. *invasa* resistance was slightly smaller in *E*. *grandis* than *E*. *tereticornis* (Table 1). In *E*. *grandis*, ANOVA indicated nonsignificant differences in *L*. *invasa* resistance among provenances and among families within provenances (Supplementary Table S2). The *h*_*i*_^2^ estimate (0.10 ± 0.02; Table 1) was low, especially compared to that of *E*. *tereticornis* (0.52 ± 0.50) calculated from the whole trial^16^. However, the *h*_*i*_^2^ for both *E*. *grandis* and *E*. *tereticornis* may be at similar magnitude considering the relatively high value of stand error shown in *E*. *tereticornis*^16^.

**Table 1:** Phenotypic characteristics for *L*. *invasa* resistance in *E*. *grandis* ‘discovery’ population and *E*. *tereticornis* ‘verification’ population. SD, standard deviation; SE, standard error.

Species	N	Mean (±SD)	Coefficient of variation (%)	*h*_*i*_^2^ (SE)
*E*. *grandis*	470	2.93 (±1.65)	56.4	0.10 (0.02)
*E*. *tereticornis*	303	3.23 (±1.58)	48.7	[0.52 (0.50)]^16^

### SSR marker polymorphism, LD and population structure

**Figure 2:**
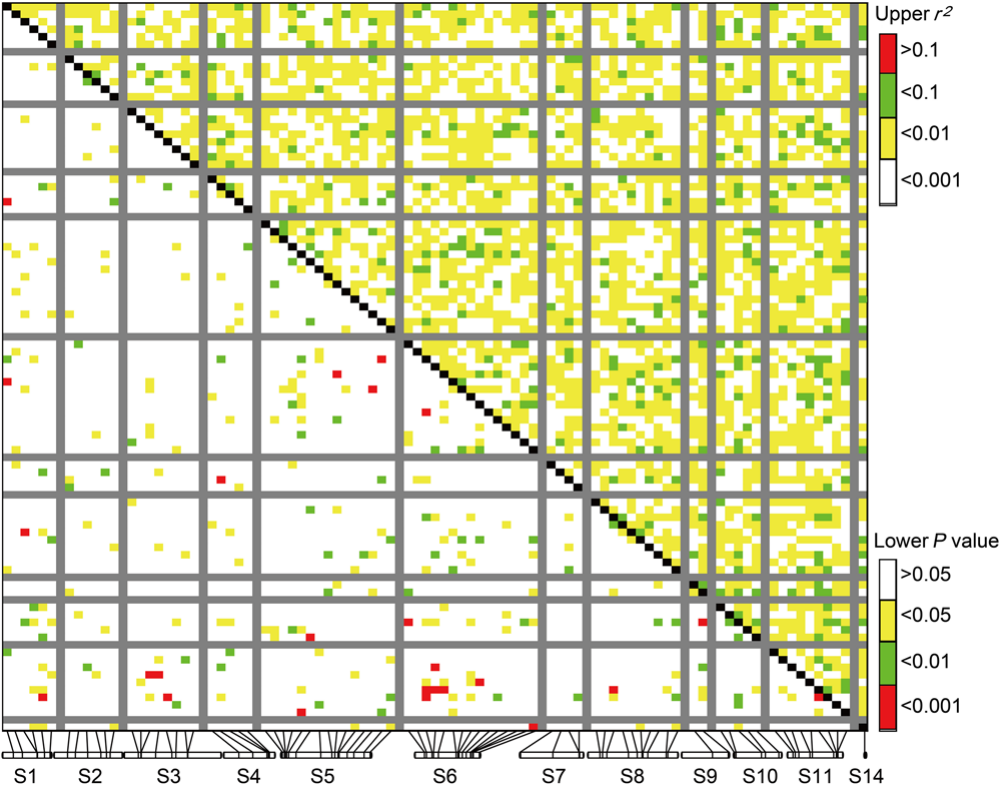
Distribution of LD (*r*^2^ value) among 86 SSR markers in *E*. *grandis*. SSR markers and their original scaffolds are along the X-axis. Each pixel above the diagonal represents the *r*^2^ value size (color code as shown in the upper right) of an SSR marker pair, and each pixel below the diagonal represents the *P* value size (color code as shown in the lower right) for testing the LD.

A total of 1,644 alleles were detected at the 86 SSR markers selected for association mapping in the *E*. *grandis* ‘discovery’ population. Polymorphic parameters differed markedly among markers, with *N*_A_ ranging from three to 60 (mean 19.1), *H*_O_ from 0.1608 to 0.9893 (mean 0.6305), *H*_E_ from 0.2049 to 0.9694 (mean 0.7771) and *PIC* from 0.1847 to 0.9673 (mean 0.7549; Supplementary Table S3). The level of LD between the 86 SSRs inferred from *E*. *grandis* was generally low, with *r*^2^ from 0 to 0.0878 (mean 0.0033) and only 87 (2.4%; *P* < 0.01) of the pairwise correlations showing significant LD (Fig. 2). Significant LD existed between linked and/or unlinked markers (Fig. 2).

Out-crossing plant species including eucalypts are expected to show a lower extent of LD compared to selfing plants^21^. The LD detected here in *E*. *grandis* is much lower than those estimated earlier in *Eucalyptus*. For example, Arumugasundaram *et al*.^59^ reported *r*^2^ values of 0−0.133 (mean 0.09) and 0−0.62 (mean 0.012) in 40 *E*. *camaldulensis* Dehnh. and 50 *E*. *tereticornis* trees, respectively, based on 62 SSRs, and Silva-Junior and Grattapaglia^31^ reported average genome-wide *r*^2^ of 0.131 in 48 *E*. *grandis* trees (two provenances) based on 21,351 SNPs. As population background can affect LD^39^, the lower LD level observed in this study could be mostly due to the larger size of population analysed (range-wide plant materials). Also, the extent of LD could vary with marker (genomic) loci^39^. Consequently, in light of a lower LD, a higher resolution of marker-trait associations can be expected.

**Figure 3:**
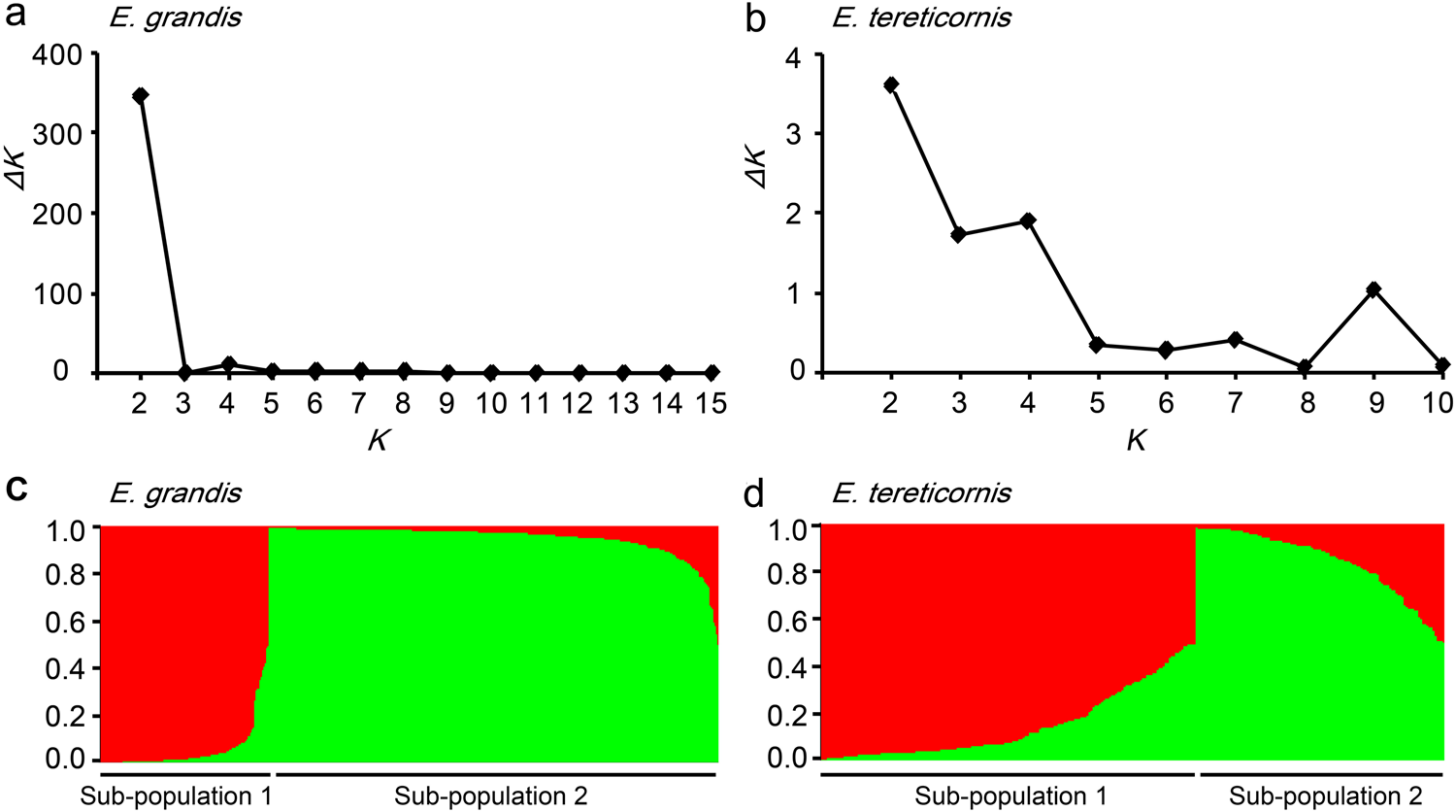
Two sub-populations inferred for 470 *E*. *grandis* and 303 *E*. *tereticornis* trees based on 25 and 12 putatively neutral genomic SSR markers, respectively. Optimal *K* value was two as determined from *ΔK* change with *K* in (**a**) *E*. *grandis* and (**b**) *E*. *tereticornis*. Two sub-populations were then partitioned with membership coefficient (Q) for each of (**c**) *E*. *grandis* and (**d**) *E*. *tereticornis*.

Population structure analysis indicated that the optimal *K* value was determined to be two for the *E*. *grandis* ‘discovery’ population (Fig. 3a), which was in agreement with previous PCA analysis on the same population^43^. The 470 individuals were thus divided into two sub-populations (Fig. 3c). Similarly, the 303 individuals of the *E*. *tereticornis* ‘verification’ population were also divided into two sub-populations (Fig. 3b and d). These results corroborate the previous division of two genetically distinct clusters of natural populations in *E*. *grandis*^43^ and *E*. *tereticornis*^44^, indicating weak genetic structure among provenances for both species. Moreover, population structure can result in spurious marker-trait relations in subsequent association mapping^38^, and the appropriate identification of genetic structure, though weak in our cases, will help to eliminate false marker-trait associations.

### Association mapping and verification

**Figure 4:**
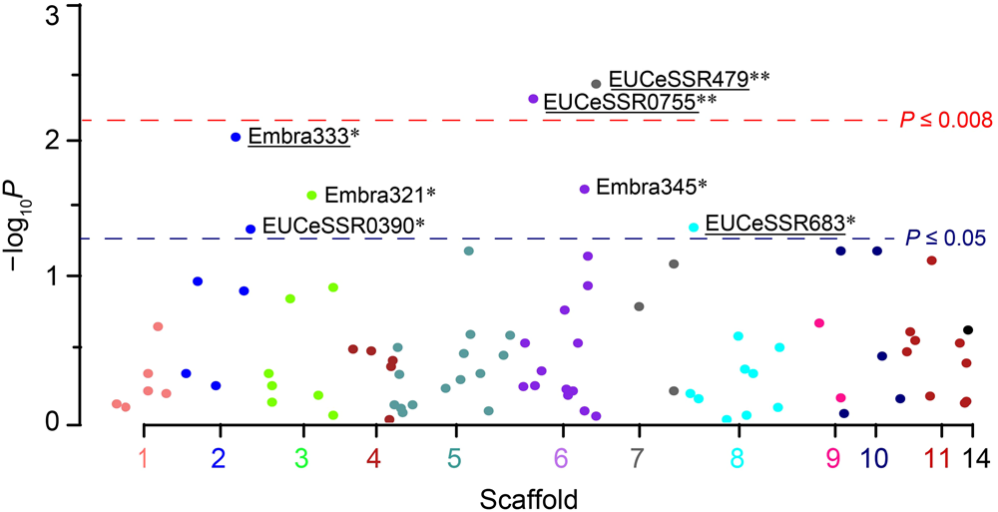
Distribution of significance levels for the 86 SSRs tested for association with *L*. *invasa* resistance in *E*. *grandis*. SSRs and their original scaffolds are along the X-axis. *P* values were transformed as −log_10_*P* (Y-axis). *P ≤ 0.05; **P ≤ 0.01 with validation (*P* ≤ 0.008) in a correction of permutation test. Four markers (underlined) were verified to be significant in *E*. *tereticornis*.

There were seven SSR markers associated in *E*. *grandis* with *L*. *invasa* resistance at the *P* ≤ 0.05 significance level, of which two (EUCeSSR0755 and EUCeSSR479) were validated with a correction of permutation test (*P* ≤ 0.008; Fig. 4, Table 2 and Supplementary Table S4). The *R*^2^ value of a significant marker ranged from 3.3% (EUCeSSR0930) to 37.8% (Embra333), with an average of 16.7%. The seven SSRs resided on scaffolds 2, 3, 6, 7, 8 and/or 5 of the *E*. *grandis* genome (Table 2). Further, four of the seven significant associations were verified and also validated in *E*. *tereticornis* (*P* ≤ 0.073 in permutation test; Table 2), with *R*^2^ ranging from 24.3% to 48.5% (averaging at 34.3%). All of the verified markers had consistently higher *R*^2^ in *E*. *tereticornis*. High *R*^2^ values have been demonstrated for SSR markers in other plants, e.g. the highest being 80% for yellow mosaic virus disease resistance in soybean^38^ and 20% for aluminum tolerance in rice^34^. The high *R*^2^ may be attributable to the multiple alleles of an SSR, of which the effect of each allele, usually small, can be accumulated to an extraordinarily high level.

**Table 2:** Seven SSRs associated significantly (*P* ≤ 0.05) with *L*. *invasa* resistance in *E*. *grandis*, of which four were verified in *E*. *tereticornis*. ^†^Significance validated with a correction of permutation test (*P* ≤ 0.008 and 0.073 for *E*. *grandis* and *E*. *tereticornis*, respectively). ^‡^Approximated as significance at *P* ≤ 0.05. *R*^2^, the percentage of phenotypic variance explained; AAE, average allelic effect.

Marker	Scaffold	*E*. *grandis*				*E*. *tereticornis*			
		*P* value	*R*^2^ (%)	Positive AAE	Negative AAE	*P* value	*R*^2^ (%)	Positive AAE	Negative AAE
Embra333	2	0.011	37.8	0.37	−0.63	0.002^†^	48.5	0.48	−0.48
EUCeSSR0930	2 (5)	0.046	3.3	0.43	−0.81	0.185	6.5	—	—
Embra321	3	0.027	8.6	—	−0.33	0.126	15.4	—	—
EUCeSSR0755	6	0.006^†^	9.1	—	−1.44	0.051^†‡^	27.3	0.35	−0.29
Embra345	6	0.024	21.3	0.85	−0.33	0.078	41.5	—	—
EUCeSSR479	7	0.005^†^	14.9	0.50	−0.35	0.022^†^	24.3	0.56	−0.10
EUCeSSR683	8	0.045	21.8	1.12	−0.47	0.043^†^	37.2	0.57	−0.19

Little is known about the genomic loci associated with response to gall wasps in plant species, and the significant markers identified here would therefore provide insight into the genetic control of insect resistance in plants. In addition to the low *h*_*i*_^2^ estimate, multiple significantly associated markers suggest the quantitative inheritance of gall wasp resistance in *Eucalyptus*. Of the seven significant loci detected in *E*. *grandis*, five (Embra333, EUCeSSR0930, Embra321, EUCeSSR479 and EUCeSSR683) are homologous to known genes or predicted proteins when their original sequences were BlastX searched against the NCBI non-redundant protein database. The locus Embra333 is functionally annotated as a C2H2 zinc finger protein (*Cynara cardunculus* var. *scolymus* L.; 9e−17 and 64% in *E*-value and similarity, respectively). C2H2 zinc finger proteins are one of the largest transcript factor families in plants and have been found to participate in diverse signal transduction pathways and developmental processes, including pathogen defense and stress responses^60^. EUCeSSR0930 has homology to M-phase inducer phosphatase 3 (*Anthurium amnicola* Dressler; 5e−22 and 70% in *E*-value and similarity, respectively), a protein that can be phosphorylated and activated by Cdkl/cyclin B and leads to entry into mitosis^61^. Embra321 is homologous to a gene annotated as predicted U-box domain-containing protein 51-like (*E*. *grandis*; 1e−8 and 85% in *E*-value and similarity, respectively). Though the physiological function of U-box domain remains unclear, plant U-box proteins have been implicated as regulators of fundamental cellular processes related to signal transduction, damage responses and programmed cell death as well as defense against biotic and abiotic stresses^62^. EUCeSSR479 is functionally related to membrane-anchored endo-1,4-beta-glucanases (*Gossypium hirsutum* L.; 8e−79 and 86% in *E*-value and similarity, respectively), which are involved in cellulose biosynthesis in plants^63^. Also, genes encoding endo-1,4-beta-glucanases have been found in bacteria, fungi, nematodes and insects. In the crown gall-forming bacterium *Agrobacterium tumefaciens*, cellulose fibers can be produced to adhere to plant cell walls during infection^64^. EUCeSSR683 is a predicted proline-rich receptor-like protein kinase PERK9 (*E*. *grandis*; 3e−22 and 98% in *E*-value and similarity, respectively), which is expressed widely in *Arabidopsis thaliana* L. Heynh.^65^ and may act as a sensor/receptor in plants to monitor changes at cell walls during cell expansion or during exposure to abiotic/biotic stresses and then activate associated cellular responses^66^. However, the remaining two loci (EUCeSSR0755 and Embra345) were of unknown function. As EUCeSSR0755 is derived from an expressed sequence tag (ES589368), it may be a CG for physiological response to gall wasp infection. Nevertheless, causal gene(s) might be located in the LD region of a significant marker locus as neutral markers may represent artificial association caused by genetic hitchhiking^67^.

Few associations in plants have been verified in a different species though several studies have verified association results in an additional full-sib mapping population of the same species, such as yellow mosaic virus resistance associated SSRs in *G*. *max*^38^ and wood-property associated SSRs in *Populus tomentosa* Carr.^68^. In our study, four of the seven significant markers in *E*. *grandis* were verified in *E*. *tereticornis*, suggesting the effectiveness of these marker-trait associations across species. However, three SSR markers remained non-significant in verification analysis with *E*. *tereticornis*. Several factors are possible to affect the verification results, including species, environment, population size and phenotyping. Related species may contain different loci affecting such a complex trait as insect resistance, which could have evolved independently in different populations and habitats^69^. Moreover, low LD in forest trees can give rise to inconsistent marker-trait associations among genotypes even within the same species^70^. Also, coupled with environment and population size, phenotyping technique plays an important role in finding accurate genotype-phenotype associations^25^. In the present study, phenotyping of gall wasp resistance was different between the ‘discovery’ and ‘verification’ species in terms of trial site, measurement age, season, population size and phenotyping method, which could be attributable, at least in part, to those non-verified associations in *E*. *tereticornis*. As the factors mentioned above are concerned, further efforts need to carry out multiple-site experiments deploying a large amount of clonally propagated genotypes.

### Mining for favourable alleles and implications for practical breeding

The alleles with positive effects are considered as favourable alleles for *L*. *invasa* resistance. Table 3 shows the first allele with the largest (positive and negative) resistance effect of each of the significant SSR markers (see Supplementary Table S5 for the effects of all the alleles). In *E*. *grandis*, the allele Embra345–225 bp had the maximum positive effect (1.67, 83.3%), whereas EUCeSSR0755-274 bp had the maximum negative effect (−1.70, 39.2%; Table 3). In *E*. *tereticornis*, the alleles EUCeSSR683-167 bp and Embra333-250 bp had the maximum positive (1.38, 47.0%) and negative (−1.45, 49.2%) effects, respectively (Table 3). In particular, some of the alleles had the opposite phenotypic effect between the two eucalypt species. For EUCeSSR479 for example, the alleles 204, 231, 213 and 222 bp showed a negative effect in *E*. *grandis* (Supplementary Table S5) but a positive effect in *E*. *tereticornis* (Supplementary Table S6), in spite of the consistently positive effect of 228, 234, 210 and 225 bp in both species. Similarly, Dillon *et al*.^17^ found a flip in the effect of a SNP on pulp yield between discovery and verification populations in the eucalypt species *Corymbia citriodora* (Hook.) K.D. Hill & L.A.S. Johnson. Such reversals in allelic effect can be a reflection of complex associations in which interactions between the associated allele and other factors are at play^17^.

**Table 3:** The first allele with the largest (positive and negative) effect on *L*. *invasa* resistance for each of the significant SSR markers in *E*. *grandis* and *E*. *tereticornis*. PVE, phenotypic variation explained; Carriers, trees carrying a specific allele.

Species	Marker	The positive effect				The negative effect			
		Allele (bp)	Effect	PVE (%)	No. carriers	Allele (bp)	Effect	PVE (%)	No. carriers
*E*. *grandis*	Embra333	214	0.79	29.0	6	250	−0.86	31.8	20
	EUCeSSR0930	208	0.43	14.3	7	205	−1.67	55.6	3
	Embra321	222	0.07	2.4	37	220	−0.65	21.3	282
	EUCeSSR0755	—	—	—	—	274	−1.70	39.2	30
	Embra345	225	1.67	83.3	15	223	−0.33	16.7	15
	EUCeSSR479	228	0.75	25.3	61	216	−1.11	37.7	12
	EUCeSSR683	161	1.12	33.0	4	163	−0.79	23.5	22
*E*. *tereticornis*	Embra333	212	0.96	32.5	11	250	−1.45	49.2	6
	EUCeSSR0755	224	0.74	23.9	14	226	−0.61	19.7	6
	EUCeSSR479	234	0.80	27.9	17	201	−0.14	4.8	7
	EUCeSSR683	167	1.38	47.0	13	143	−0.56	19.0	8

This study reveals markers and favourable alleles that have potential in marker-assisted selection for resistance to the gall wasp *L*. *invasa*, at least in the important tree genus *Eucalyptus*. Firstly, the significant markers, especially those validated, could be used to track genomic loci of interest. While the SSRs associated in *E*. *grandis* provide candidates for verification purpose in other species, the associations verified in *E*. *tereticornis* may suggest robust genomic regions underlying gall wasp resistance. Secondly, the favourable alleles could be used as tags of selection for elite genotypes that have assembled the most desirable alleles over all the associated markers. This is extremely useful for species (e.g. many eucalypts) amenable to mass vegetative propagation as clonal cultivation offers the most effective approach to capture genetic gains^71^. Thirdly, based on the association results, elite parental combinations could be predicted to generate descendants with improved resistance to *L*. *invasa*. In this respect, the alleles robust in both *E*. *grandis* and *E*. *tereticornis* will have greater utility in a breeding programme.

In conclusion, this study presents genomic loci associated with gall-inducing insect resistance for the first time in plants and makes a valuable contribution to our understanding of the genetic basis underlying plant resistance to gall wasps. Seven SSR markers were associated with resistance to *L*. *invasa* in *E*. *grandis*, of which four associations were verified in *E*. *tereticornis*. These markers plus their favourable alleles can be used for marker-assisted selection for *L*. *invasa* resistance in *Eucalyptus*. Nevertheless, considering the quantitative nature of the insect resistance and the small proportion of genome sampled by the SSR loci, further association work should be undertaken to improve the genome coverage of markers by applying new technologies, such as next-generation sequencing^72^. In addition, as CG approaches have proven to be advantageous for breeding applications^17^, genome-wide screening of CGs followed by CG-based association mapping should be conducted for gall wasp resistance.

## Electronic supplementary material

Supplementary Figure S1 and Tables S1–S6
