# Supplementary material for: The first identification of genomic loci in plants associated with resistance to galling insects: a case study in Eucalyptus L'Hér. (Myrtaceae)
Source: Sci Rep. 2018 Feb 2;8:2319. doi: 10.1038/s41598-018-20780-9 (PMC5797152; doi:10.1038/s41598-018-20780-9)
Supplement: Supplementary file 1 — Supplementary Figure S1 and Tables S1–S6 [file 41598_2018_20780_MOESM1_ESM.pdf]

Supplementary Information

The first identification of genomic loci in plants associated with resistance to galling insects: a case study in *Eucalyptus* L'Hér. (Myrtaceae)

Miaomiao Zhang[1,2,3,†], Changpin Zhou[1,2,†], Zhijiao Song[1,2,4,†], Qijie Weng[2], Mei Li[2], Hongxia Ji[2], Xiaoyong Mo[3], Huanhua Huang[5], Wanhong Lu[6], Jianzhong Luo[6], Fagen Li[1,2,*] & Siming Gan[1,2,*]

[1]State Key Laboratory of Tree Genetics and Breeding, Chinese Academy of Forestry, Xiangshan Road, Beijing 100091, China. [2]Key Laboratory of State Forestry Administration on Tropical Forestry Research, Research Institute of Tropical Forestry, Chinese Academy of Forestry, Longdong, Guangzhou 510520, China. [3]College of Forestry, South China Agricultural University, 284 Block, Wushan Street, Guangzhou 510642, China. [4]Baoshan University, Yuanzheng Road, Baoshan 678000, China, [5]Guangdong Academy of Forestry, Longdong, Guangzhou 510520, China. [6]China Eucalypt Research Centre, Zhanjiang 524022, China. [†]These authors contributed equally to this work. [*]Correspondence and requests for materials should be addressed to F.L. (email: lifagen2002@126.com) or S.G. (email: siming.gan@ritf.ac.cn)

Supplementary Figure S1 and Tables S1–S6

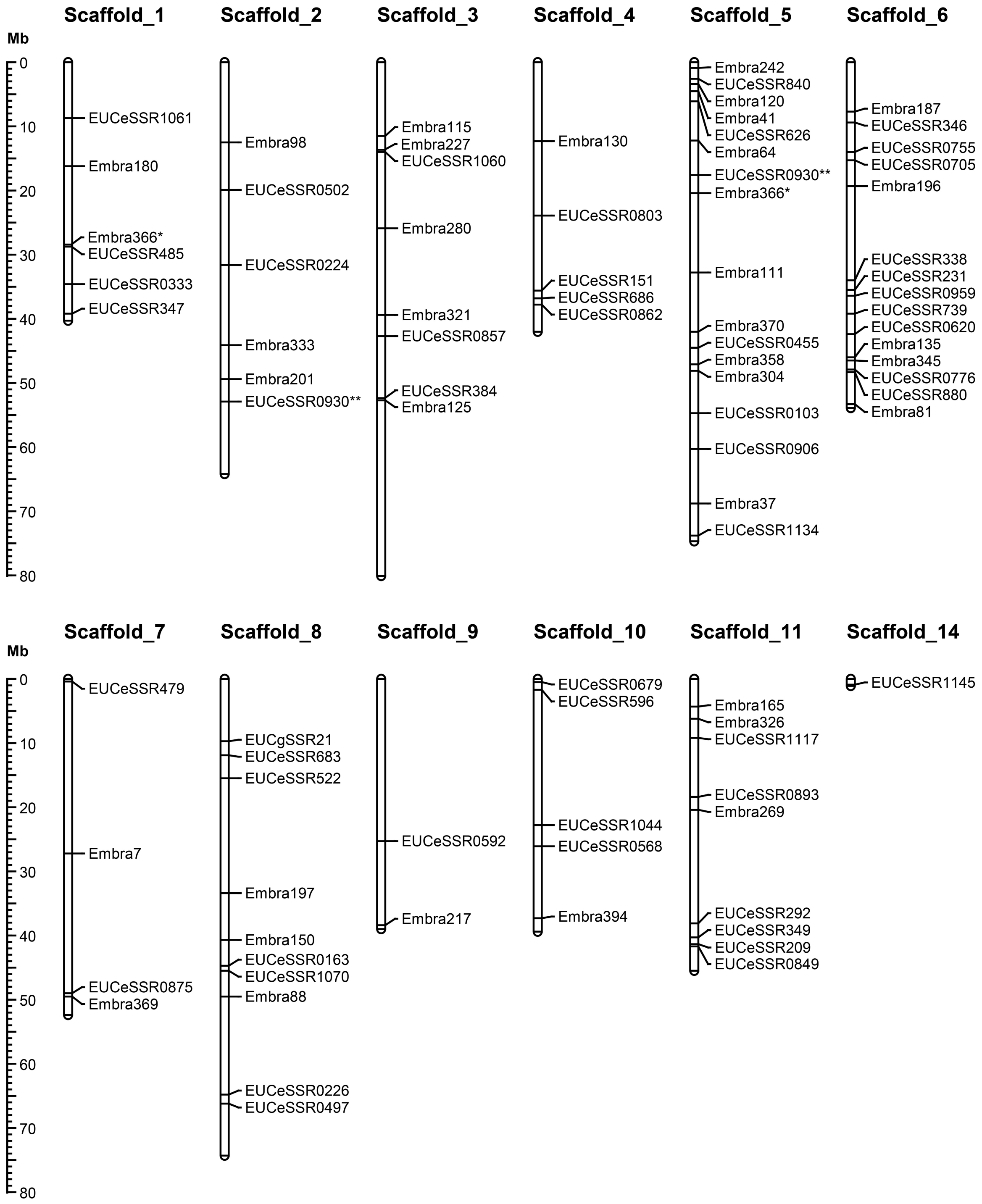

Supplementary Figure S1. Physical location of the 86 SSR markers selected out for association mapping in *E. grandis* 'discovery' populations. Marker sequences were aligned onto the *E. grandis* genome assembly v1.1 (http://www.phytozome.net/eucalyptus.php). Two markers denoted with asterisks (* or **) have duplicate positions, namely, Embra366 on scaffolds 1 and 5 and EUCeSSR0930 on scaffolds 2 and 5.

Supplementary Table S1. Twenty-five and twelve putatively neutral genomic SSR markers used for population structure analysis in *E. grandis* and *E. tereticornis*, respectively

SSR marker[a]	Scaffold[b]	Serial no. for *E. grandis*	Serial no. for *E. tereticornis*
Embra180	1	1	
Embra366	1 (5)	2	
Embra333	2	3	1
Embra201	2	4	
Embra227	3	5	
Embra280	3		2
Embra321	3	6	
Embra125	3	7	
Embra130	4	8	3
Embra120	5		4
Embra41	5	9	
Embra64	5	10	5
Embra111	5	11	
Embra370	5	12	
Embra358	5	13	
Embra188	5		6
Embra37	5	14	
Embra196	6	15	
Embra135	6	16	
Embra345	6	17	
Embra81	6	18	
Embra7	7	19	
Embra369	7	20	
Embra197	8		7
Embra150	8	21	
Embra88	8	22	8
Embra217	9		9
Embra394	10		10
Embra87	11		11
Embra326	11	23	
Embra269	11	24	12
Embra258	11	25	

[a] Markers prefixed with Embra were developed by Brondani et al[1].

[b] Scaffold was determined with aligning a marker sequence to *E. grandis* genome assembly (version 1.1, http://www.phytozome.net/eucalyptus.php).

Supplementary Table S2. Analysis of variance (ANOVA) of *L. invasa* resistance in *E. grandis* 'discovery' population

Source	Degree of freedom	Mean square	F value	*Pr > F*
Among replicates[a]	3	5.30	2.07	0.11
Among provenances	15	3.19	1.25	0.24
Among families within provenance	137	2.94	1.15	0.18
Residual error	209	2.55		

[a] Based on the relatively complete replicates 1–4.

Supplementary Table S3. Genetic parameters for the 86 SSR markers tested in *E. grandis*

No.	SSR marker[a]	N_A	Allele size range (bp)	H_O	H_E	*PIC*
1	EUCeSSR1061	20	234–276	0.6407	0.7248	0.7061
2	Embra180	22	100–150	0.7570	0.9096	0.9026
3	Embra366	28	86–144	0.7253	0.8623	0.8514
4	EUCeSSR485	9	136–184	0.7521	0.7552	0.7180
5	EUCeSSR0333	21	99–145	0.8110	0.7991	0.7760
6	EUCeSSR347	38	128–208	0.5802	0.9620	0.9595
7	Embra98	27	202–268	0.7106	0.9366	0.9319
8	EUCeSSR0502	12	285–324	0.7756	0.6080	0.5459
9	EUCeSSR0224	13	93–117	0.9756	0.7867	0.7566
10	Embra333	22	214–256	0.8078	0.9286	0.9231
11	Embra201	19	126–162	0.4888	0.8582	0.8430
12	EUCeSSR0930	7	202–223	0.3134	0.2882	0.2617
13	Embra115	19	94–130	0.3618	0.8612	0.8485
14	Embra227	20	291–331	0.7262	0.8815	0.8698
15	EUCeSSR1060	5	164–176	0.2076	0.5980	0.5372
16	Embra280	29	75–141	0.2852	0.9264	0.9201
17	Embra321	9	212–250	0.5443	0.6208	0.5489
18	EUCeSSR0857	17	139–173	0.7897	0.8738	0.8599
19	EUCeSSR384	22	104–150	0.9046	0.9009	0.8915
20	Embra125	29	137–201	0.8688	0.9394	0.9350
21	Embra130	26	88–144	0.6967	0.9163	0.9096
22	EUCeSSR0803	21	199–241	0.4718	0.6080	0.5902
23	EUCeSSR151	5	442–450	0.5777	0.5996	0.5158
24	EUCeSSR686	17	184–220	0.8614	0.8789	0.8667
25	EUCeSSR0862	21	222–272	0.8106	0.7440	0.7282
26	Embra242	39	122–202	0.7235	0.9520	0.9489
27	EUCeSSR840	8	199–215	0.6867	0.7840	0.7502
28	Embra120	22	128–170	0.7725	0.8997	0.8903
29	Embra41	5	232–252	0.8261	0.5697	0.4939
30	EUCeSSR626	10	193–223	0.9847	0.6736	0.6182
31	Embra64	22	172–214	0.8022	0.9096	0.9024
32	Embra111	42	98–190	0.7209	0.9372	0.9328
33	Embra370	19	111–167	0.5398	0.7793	0.7503
34	EUCeSSR0455	9	82–102	0.5577	0.6701	0.6165
35	Embra358	15	118–148	0.5708	0.7591	0.7307

No.	SSR marker[a]	N_A	Allele size range (bp)	H_O	H_E	*PIC*
36	Embra304	60	182–258	0.4202	0.9694	0.9673
37	EUCeSSR0103	27	168–220	0.6699	0.9111	0.9035
38	EUCeSSR0906	12	196–253	0.7543	0.7045	0.6800
39	Embra37	32	104–168	0.7687	0.9316	0.9265
40	EUCeSSR1134	19	131–167	0.7247	0.8607	0.8454
41	Embra187	33	140–214	0.5986	0.8838	0.8741
42	EUCeSSR346	25	221–273	0.5952	0.9099	0.9025
43	EUCeSSR0755	10	266–284	0.4787	0.7160	0.6754
44	EUCeSSR0705	3	293–299	0.1608	0.2049	0.1847
45	Embra196	34	202–284	0.4624	0.6451	0.6285
46	EUCeSSR338	15	216–267	0.3952	0.7093	0.6683
47	EUCeSSR231	6	110–131	0.5355	0.4311	0.3886
48	EUCeSSR0959	8	124–145	0.2263	0.2587	0.2471
49	EUCeSSR739	16	238–316	0.5676	0.7462	0.7012
50	EUCeSSR0620	12	105–147	0.8593	0.6819	0.6294
51	Embra135	24	143–189	0.8044	0.8782	0.8662
52	Embra345	26	195–251	0.8657	0.8492	0.8337
53	EUCeSSR0776	4	131–149	0.4191	0.4158	0.3867
54	EUCeSSR880	28	355–413	0.6022	0.8413	0.8217
55	Embra81	25	77–131	0.8280	0.8656	0.8517
56	EUCeSSR479	15	198–243	0.7950	0.7874	0.7649
57	Embra7	31	121–183	0.8201	0.9431	0.9391
58	EUCeSSR0875	9	85–115	0.9893	0.7160	0.6722
59	Embra369	21	81–145	0.7591	0.8806	0.8700
60	EUCgSSR21	13	185–230	0.6084	0.7524	0.7216
61	EUCeSSR683	20	137–175	0.7216	0.8824	0.8705
62	EUCeSSR522	18	255–291	0.6681	0.8628	0.8481
63	Embra197	21	249–289	0.8759	0.9154	0.9083
64	Embra150	15	113–145	0.7122	0.7410	0.7049
65	EUCeSSR0163	23	222–266	0.8130	0.9071	0.8986
66	EUCeSSR1070	21	214–256	0.5752	0.9007	0.8911
67	Embra88	36	91–169	0.5485	0.7600	0.7492
68	EUCeSSR0226	5	291–309	0.4211	0.5000	0.4607
69	EUCeSSR0497	8	153–173	0.9660	0.7471	0.7023
70	EUCeSSR0592	11	205–244	0.4919	0.6546	0.6321
71	Embra217	20	108–150	0.6538	0.9067	0.8984

No.	SSR marker[a]	N_A	Allele size range (bp)	H_O	H_E	*PIC*
72	EUCeSSR0679	21	304–330	0.2848	0.7284	0.7130
73	EUCeSSR596	20	320–362	0.6602	0.8841	0.8732
74	EUCeSSR1044	8	400–442	0.2270	0.2920	0.2790
75	EUCeSSR0568	21	86–128	0.4846	0.8491	0.8316
76	Embra394	19	205–245	0.3680	0.8861	0.8746
77	Embra165	25	92–144	0.8404	0.9351	0.9302
78	Embra326	11	222–244	0.5915	0.7022	0.6670
79	EUCeSSR1117	19	123–165	0.3889	0.7695	0.7388
80	EUCeSSR0893	19	407–530	0.2790	0.8580	0.8429
81	Embra269	23	187–233	0.6918	0.8807	0.8689
82	EUCeSSR292	13	324–348	0.2201	0.8056	0.7815
83	EUCeSSR349	28	299–353	0.6681	0.8030	0.7828
84	EUCeSSR209	6	298–308	0.5551	0.6790	0.6334
85	EUCeSSR0849	13	212–238	0.4206	0.8596	0.8442
86	EUCeSSR1145	23	107–155	0.7466	0.9237	0.9174
Total		1644				
Mean (SD)		19.12 (9.83)		0.6305 (0.2013)	0.7771 (0.1675)	0.7549 (0.1775)

N_A, number of alleles; H_O, observed heterozygosity; H_E, expected heterozygosity; *PIC*, polymorphic information content; SD, standard deviation.

[a] Markers prefixed with Embra were developed by Brondani *et al*[1]. Markers with prefix EUCeSSR followed by three and four numerals were developed by He *et al*[2]. and Zhou *et al*[3], respectively, except EUCeSSR1060 developed by Zhou *et al*[4]. EUCgSSR21 was developed by He *et al*[5].

Supplementary Table S4. The significance level (*P* value) and the percentage of phenotypic variance explained (R^2) for the 86 SSRs tested for association with *L. invasa* resistance in *E. grandis*

No.	SSR marker[a]	*P* value	$-\log_{10} P$	R^2 (%)
1	EUCeSSR1061	0.760	0.119	11.6
2	Embra180	0.769	0.114	24.0
3	Embra366	0.465	0.333	28.7
4	EUCeSSR485	0.621	0.207	5.0
5	EUCeSSR0333	0.220	0.658	13.8
6	EUCeSSR347	0.648	0.189	34.5
7	Embra98	0.467	0.330	33.2
8	EUCeSSR0502	0.108	0.968	7.3
9	EUCeSSR0224	0.569	0.245	6.9
10	Embra333*	0.011	1.972	37.8
11	Embra201	0.125	0.904	21.0
12	EUCeSSR0930*	0.046	1.334	3.3
13	Embra115	0.468	0.329	17.5
14	Embra227	0.565	0.248	21.7
15	EUCeSSR1060	0.736	0.133	1.5
16	Embra280	0.141	0.852	29.0
17	Embra321*	0.027	1.569	8.6
18	EUCeSSR0857	0.662	0.179	11.3
19	EUCeSSR384	0.914	0.039	15.1
20	Embra125	0.118	0.928	40.7
21	Embra130	0.317	0.499	31.2
22	EUCeSSR0803	0.325	0.488	12.9
23	EUCeSSR151	0.978	0.010	0.6
24	EUCeSSR686	0.420	0.376	12.5
25	EUCeSSR0862	0.381	0.419	11.7
26	Embra242	0.773	0.112	27.2
27	EUCeSSR840	0.307	0.512	6.5
28	Embra120	0.473	0.325	23.6
29	Embra41	0.819	0.087	1.0
30	EUCeSSR626	0.877	0.057	2.2
31	Embra64	0.769	0.114	26.9
32	Embra111	0.595	0.226	33.4
33	Embra370	0.512	0.291	12.1
34	EUCeSSR0455	0.339	0.470	5.5
35	Embra358	0.066	1.182	11.6
36	Embra304	0.249	0.605	45.1

No.	SSR marker[a]	P value	$-\log_{10} P$	R^2 (%)
37	EUCeSSR0103	0.471	0.327	20.7
38	EUCeSSR0906	0.846	0.073	6.3
39	Embra37	0.349	0.457	38.4
40	EUCeSSR1134	0.252	0.598	17.1
41	Embra187	0.573	0.242	24.9
42	EUCeSSR346	0.288	0.541	28.0
43	EUCeSSR0755**	0.006	2.237	9.1
44	EUCeSSR0705	0.570	0.244	0.5
45	Embra196	0.451	0.346	17.3
46	EUCeSSR338	0.169	0.772	7.8
47	EUCeSSR231	0.598	0.223	1.2
48	EUCeSSR0959	0.664	0.178	2.1
49	EUCeSSR739	0.616	0.210	5.7
50	EUCeSSR0620	0.287	0.543	5.4
51	Embra135	0.851	0.070	15.7
52	Embra345*	0.024	1.614	21.3
53	EUCeSSR0776	0.115	0.940	2.2
54	EUCeSSR880	0.071	1.149	23.4
55	Embra81	0.925	0.034	14.6
56	EUCeSSR479**	0.005	2.343	14.9
57	Embra7	0.159	0.798	39.5
58	EUCeSSR0875	0.620	0.208	2.5
59	Embra369	0.081	1.092	24.1
60	EUCgSSR21	0.642	0.192	6.9
61	EUCeSSR683*	0.045	1.344	21.8
62	EUCeSSR522	0.703	0.153	14.6
63	Embra197	0.982	0.008	19.7
64	Embra150	0.258	0.588	10.4
65	EUCeSSR0163	0.436	0.361	16.0
66	EUCeSSR1070	0.916	0.038	15.5
67	Embra88	0.468	0.329	24.9
68	EUCeSSR0226	0.807	0.093	1.4
69	EUCeSSR0497	0.309	0.510	2.5
70	EUCeSSR0592	0.209	0.680	10.6
71	Embra217	0.694	0.159	21.4
72	EUCeSSR0679	0.065	1.184	14.7
73	EUCeSSR596	0.886	0.053	15.9
74	EUCeSSR1044	0.066	1.181	4.6
75	EUCeSSR0568	0.354	0.451	16.5

No.	SSR marker[a]	P value	$-\log_{10} P$	R^2 (%)
76	Embra394	0.699	0.155	17.8
77	Embra165	0.330	0.482	33.4
78	Embra326	0.240	0.621	7.7
79	EUCeSSR1117	0.276	0.558	11.9
80	EUCeSSR0893	0.674	0.171	13.1
81	Embra269	0.076	1.116	24.4
82	EUCeSSR292	0.288	0.541	10.6
83	EUCeSSR349	0.750	0.125	14.7
84	EUCeSSR209	0.396	0.403	4.5
85	EUCeSSR0849	0.735	0.134	14.1
86	EUCeSSR1145	0.233	0.632	33.4

[a] Markers prefixed with Embra were developed by Brondani *et al*[1]. Markers with prefix EUCeSSR followed by three and four numerals were developed by He *et al*[2]. and Zhou *et al*[3], respectively, except EUCeSSR1060 developed by Zhou *et al*[4]. EUCgSSR21 was developed by He *et al*[5]. *, $P \leq 0.05$; **, $P \leq 0.01$ with validation ($P \leq 0.008$, underlined) in a correction of permutation test.

Supplementary Table S5. The allelic positive and negative effect on *L. invasa* resistance for each of the significant SSR markers in *E. grandis*

Marker	The positive effect				The negative effect			
	Allele (bp)	Effect	PVE (%)	No. carriers[a]	Allele (bp)	Effect	PVE (%)	No. carriers[a]
Embra333	214	0.79	29.0	6	250	−0.86	31.8	20
	238	0.74	27.3	11	252	−0.64	23.5	13
	254	0.69	25.3	15	218	−0.55	20.2	12
	256	0.57	21.1	7	248	−0.45	16.6	19
	234	0.54	19.7	40				
	228	0.52	19.1	30				
	220	0.42	15.3	46				
	216	0.38	13.8	89				
	226	0.33	12.3	61				
	240	0.29	10.5	96				
	222	0.28	10.2	104				
	244	0.22	8.0	74				
	242	0.21	7.7	26				
	246	0.20	7.3	45				
	232	0.19	7.2	22				
	236	0.19	7.0	31				
	224	0.07	2.5	32				
	230	0.05	1.8	38				
EUCeSSR0930	208	0.43	14.3	7	205	−1.67	55.6	3
					223	−1.33	44.4	9
					202	−0.67	22.2	9
					220	−0.31	10.4	125
					217	−0.06	1.9	126
Embra321	222	0.07	2.4	37	220	−0.65	21.3	282
	226	0.06	1.9	25	224	−0.34	11.0	316
					234	−0.19	6.1	8
					232	−0.14	4.5	33
					212	−0.03	0.9	17
EUCeSSR0755					274	−1.70	39.2	30
					276	−1.51	34.9	280
					278	−1.51	34.8	179
					266	−1.47	33.8	54

Marker	The positive effect				The negative effect			
	Allele (bp)	Effect	PVE (%)	No. carriers[a]	Allele (bp)	Effect	PVE (%)	No. carriers[a]
					280	−1.37	31.5	92
					282	−1.10	25.3	59
Embra345	225	1.67	0.8	15	223	−0.33	16.7	15
	243	1.54	0.8	24				
	237	1.50	0.8	6				
	231	1.35	0.7	20				
	227	1.23	0.6	30				
	215	1.20	0.6	5				
	213	1.19	0.6	21				
	221	1.07	0.5	230				
	205	1.01	0.5	124				
	203	0.96	0.5	50				
	219	0.89	0.5	170				
	229	0.64	0.3	72				
	233	0.40	0.2	10				
	239	0.31	0.2	13				
	247	0.31	0.2	13				
	241	0.30	0.2	20				
	209	0.29	0.1	7				
	211	0.25	0.1	4				
	207	0.13	0.1	16				
EUCeSSR479	228	0.75	25.3	61	216	−1.11	37.7	12
	234	0.63	21.4	7	243	−0.44	15.1	4
	207	0.49	16.5	14	204	−0.34	11.4	132
	210	0.41	14.0	31	231	−0.26	8.7	51
	225	0.25	8.4	110	213	−0.21	7.1	15
					219	−0.08	2.6	292
					222	−0.01	0.4	57
EUCeSSR683	161	1.12	33.0	4	163	−0.79	23.5	22
					165	−0.70	20.7	38
					139	−0.69	20.5	58
					167	−0.65	19.2	30
					147	−0.59	17.6	43
					151	−0.52	15.5	114
					155	−0.46	13.5	55

Marker	The positive effect				The negative effect			
	Allele (bp)	Effect	PVE (%)	No. carriers[a]	Allele (bp)	Effect	PVE (%)	No. carriers[a]
					145	−0.38	11.4	17
					153	−0.38	11.1	132
					141	−0.37	11.0	161
					149	−0.37	10.9	70
					157	−0.16	4.8	9
					143	−0.04	1.2	58

PVE, phenotypic variation explained.

[a] Carriers refer to trees carrying a specific allele.

Supplementary Table S6. The allelic positive and negative effect on *L. invasa* resistance for each of the significant SSR markers in *E. tereticornis*

Marker	The positive effect				The negative effect			
	Allele (bp)	Effect	PVE (%)	No. carriers[a]	Allele (bp)	Effect	PVE (%)	No. carriers[a]
Embra333	212	0.96	32.5	11	250	−1.45	49.2	6
	208	0.94	31.8	9	254	−0.20	6.8	4
	246	0.91	30.8	7	230	−0.17	5.8	9
	244	0.83	28.1	10	226	−0.09	3.2	28
	240	0.74	25.0	16				
	214	0.68	22.9	8				
	248	0.65	22.0	5				
	224	0.65	21.9	47				
	228	0.61	20.5	18				
	234	0.48	16.4	23				
	220	0.48	16.2	35				
	222	0.23	7.9	87				
	218	0.23	7.7	34				
	232	0.18	6.1	23				
	242	0.18	6.1	23				
	216	0.18	6.1	85				
	238	0.12	4.1	14				
	236	0.10	3.4	20				
	210	0.05	1.7	6				
EUCeSSR0755	224	0.74	23.9	14	226	−0.61	19.7	6
	218	0.69	22.0	11	212	−0.45	14.4	3
	242	0.66	21.3	18	250	−0.45	14.4	3
	208	0.49	15.6	5	240	−0.17	5.5	18
	220	0.30	9.5	40	228	−0.45	3.7	3
	236	0.25	8.1	49	230	−0.45	3.7	9
	244	0.25	8.0	11	234	−0.61	3.7	78
	204	0.22	7.0	3				
	238	0.11	3.5	9				
	222	0.04	1.2	119				
	232	0.01	0.1	52				
EUCeSSR479	234	0.80	27.9	17	201	−0.14	4.8	7
	213	0.77	27.0	37	219	−0.07	2.3	56

Marker	The positive effect				The negative effect			
	Allele (bp)	Effect	PVE (%)	No. carriers[a]	Allele (bp)	Effect	PVE (%)	No. carriers[a]
	222	0.73	25.5	19				
	237	0.65	22.7	8				
	231	0.59	20.5	48				
	210	0.48	16.9	12				
	228	0.42	14.9	178				
	225	0.35	12.4	45				
	204	0.24	8.4	12				
EUCeSSR683	167	1.38	47.0	13	143	−0.56	19.0	8
	145	0.93	31.8	22	161	−0.32	10.8	36
	141	0.93	31.6	14	149	−0.12	4.0	16
	165	0.53	18.2	28	151	−0.07	2.5	61
	153	0.48	16.5	58	163	−0.07	2.5	28
	155	0.35	12.0	39	147	−0.02	0.8	11
	159	0.35	11.9	43				
	157	0.33	11.3	42				
	169	0.32	10.9	8				
	139	0.07	2.4	6				

PVE, phenotypic variation explained.

[a] Carriers refer to trees carrying a specific allele.

References

1. Brondani, R. P. V., Williams, E. R., Brondani, C. & Grattapaglia, D. A microsatellite-based consensus linkage map for species of *Eucalyptus* and a novel set of 230 microsatellite markers for the genus. *BMC Plant Biology* **6,** 20 (2006).

2. He, X. *et al*. Development of 198 novel EST-derived microsatellites in *Eucalyptus* (Myrtaceae). *Am. J. Bot.* **99,** e134–e148 (2012).

3. Zhou, C. *et al*. Development of 240 novel EST-SSRs in *Eucalyptus* L'Hérit. *Mol. Breed.* **33,** 221–225 (2014).

4. Zhou, C. *et al*. Comparison between direct sequencing and pool-cloning-based sequencing of PCR products in EST-SSR marker development in *Eucalyptus*. *Mol. Plant Breed.* **8,** e1 (2010).

5. He, X., Li, F., Shi, J. & Gan, S. Seven genomic SSR markers revealed in *Eucalyptus* by re-sequencing of DNA sequences from GenBank. *Silvae Genet.* **60,** 92–94 (2011).
